# Micro-Sprinkling Fertigation Enhances Wheat Grain Yield and Nitrogen Use Efficiency by Reducing N Redundancy and Increasing Root–Water–Nitrogen Spatiotemporal Coordination

**DOI:** 10.3390/plants14172713

**Published:** 2025-09-01

**Authors:** Mengjing Zheng, Yingjia Zhao, Lihua Zhang, Liyan Hao, Zhongyi Zhang, Lihua Lv, Jingting Zhang

**Affiliations:** 1 Institute of Cereal and Oil Crops, Hebei Academy of Agriculture and Forestry Sciences/Hebei Key Laboratory of Crop Cultivation Physiology and Green Production, Shijiazhuang 050035, China; zhengmj96@126.com (M.Z.); zyj1039172852@163.com (Y.Z.); lnzlh@126.com (L.Z.); nkyllh@163.com (L.L.); 2 Hebei Provincial General Extension Station of Agro-Technology, Shijiazhuang 050035, China; hly2004@126.com (L.H.); zzy863@sina.com (Z.Z.)

**Keywords:** winter wheat (*Triticum aestivum* L.), water–nitrogen integration, ^15^N isotope labelling, root–water–nitrogen distribution, crop productivity

## Abstract

Micro-sprinkling fertigation, a novel irrigation and fertilization way, can improve the grain yield (GY) and nitrogen use efficiency (NUE) of winter wheat to meet sustainable agriculture requirements. In order to clarify the physiological basis behind the improvements, a field experiment with a split-plot design was conducted during the 2020–2021 and 2021–2022 growing seasons. The main plot encompassed two irrigation and fertilization modes, namely, conventional irrigation and fertilization (CIF) and micro-sprinkling fertigation (MSF), and the subplots included four nitrogen application rates (0, 120, 180, and 240 kg ha^−1^, denoted as N0, N120, N180, and N240, respectively). Moreover, a ^15^N isotopic tracer experiment was performed to determine the distributions of nitrogen in the soil. Compared with those under CIF, the GY under MSF at N180 and N240 significantly increased by 9.09% and 9.72%, which was driven mainly by increases in the grain number (GN) and thousand-grain weight (TGW). The increase in the TGW under MSF was the result of the significantly increased net photosynthesis rate at the grain-filling stage. Notably, the number and dry weight of inefficient tillers and the number of ears with fewer than 10 grains were significantly lower under MSF than those under CIF. In addition, the ^15^N isotopic tracer experiment revealed that nitrogen was primarily concentrated in the 0–30 cm soil layers under MSF, which conforms well with the spatial distributions of the roots and water, and subsequently improved the NUE under N180 and N240. In conclusion, MSF enhanced both the GY and NUE at the N180 level by optimizing root–water–nitrogen spatiotemporal coordination and reducing redundant tillering.

## 1. Introduction

The North China Plain (NCP), recognized as a critical agricultural zone in China, serves as the primary production area for winter wheat, contributing approximately 60% to the total wheat production of the country [1]. Notably, only 30% of the annual precipitation occurs during the wheat growing season, while the crop water demand is exacerbated by elevated evapotranspiration rates. The substantial soil water deficit has necessitated intensive groundwater irrigation, resulting in groundwater tables decreasing by 1–2 m annually [2,3,4]. To ensure yield stabilization and increase the resource use efficiency, advanced water-saving irrigation techniques have become imperative for sustainable agricultural development.

Furrow surface flooding, which is the traditional irrigation method for winter wheat in the NCP, exhibits multiple limitations, including excessive water consumption, uneven water distribution, low irrigation efficiency, and land resource waste [5]. Concurrently, winter wheat cultivation in this region is characterized by intensive nitrogen fertilizer application, with average nitrogen inputs reaching 280 kg ha^−1^ [6]. Typically, the surface broadcasting of nitrogen fertilizer as topdressing combined with flooding irrigation significantly increases the risk of ammonia volatilization losses [7]. Excessive and unreasonable nitrogen management practices have resulted in a series of environmental problems, including water eutrophication, the nitrate contamination of groundwater, and increased greenhouse gas emissions [8,9,10]. The fertigation technology is being applied more and more widely in agricultural production. It mainly includes sprinkler irrigation and drip irrigation. Both have the advantages of water conservation, being unrestricted by terrain and labor saving. However, compared with drip irrigation, sprinkler irrigation has the disadvantages of slightly poorer water-saving and being affected by wind force. Compared with sprinkler irrigation, drip irrigation has higher requirements for water quality. However, micro-sprinkling fertigation (MSF), an innovative irrigation and fertilization technique that integrates the advantages of drip irrigation and sprinkler irrigation, has been widely adopted in wheat production across the NCP in recent years. According to the official statistics, the adoption area of water- and fertilizer-integrated technology including MSF was about 10 million hectares (FAO). This technology provides multiple benefits, including uniform and precise water application, optimized canopy microclimate conditions, substantial yield enhancement, and increased resource use efficiency [5,11,12]. However, the effects of the optimized MSF on the yield and NUE of winter wheat are still unclear, and further studies are needed.

An optimal tillering capacity is crucial for achieving sufficient spike numbers in wheat, a fundamental requirement for obtaining a high grain yield (GY). However, excessive tillering may lead to the redundant allocation of carbon (C) and nitrogen (N) to inefficient tillers, resulting in resource competition that ultimately reduces the percentage of productive tillers [13]. The tillering capacity is significantly influenced by irrigation and fertilization practices. Conventional irrigation and fertilization practices frequently result in a large number of ineffective tillers due to the excessive application of water and nutrients. Such mismanagement promotes nonproductive accumulation in straw tissues while limiting matter remobilization to developing grains, thereby constraining both the resource use efficiency and yield potential [14]. In contrast, MSF entails the employment of precision management strategies that aim to synchronize water and nutrient supplies with the crop demand across growth stages. Nevertheless, there is little information on the tillering occurrence and N accumulation, distribution, and transfer characteristics under MSF with different N application levels.

During the winter wheat growing season in the NCP, the majority of applied fertilizers is rapidly converted into NO_3_^−^-N within weeks of application [15]. Studies have demonstrated that NO_3_^−^-N leaching below the 1.0 m soil depth constitutes the primary pathway for nitrogen loss in this agricultural system [16]. This leaching process is primarily driven by fertilization and irrigation practices, with significant nitrate leaching typically occurring after irrigation, especially under water surplus conditions [17,18]. Existing intensive farming practices in the NCP, characterized by CIF management, involve substantial gaps between input doses and crop requirements. Field investigations have revealed that nitrogen application rates exceed the winter wheat demand, and irrigation amounts surpass crop evapotranspiration [6]. These excessive inputs result in notable nitrate accumulation, with soil profile analyses revealing significant NO_3_^−^-N enrichment below the 90 cm depth [6]. In addition, the nitrogen application stage had a significant effect on the physiological characters in winter wheat. Postponing and reducing basal N fertilization can maintain high yields and improve the NUE by increasing the leaf area index, enhancing the flag leaf photosynthetic rate, and boosting the activities of nitrate reductase and glutamine synthase in flag leaves [19]. However, the physiological mechanisms by which MSF enhances the wheat yield and efficiency through the precise regulation of the irrigation/fertilization timing and amount need further elucidation.

Root systems, as vital plant organs, play crucial roles in the acquisition of water and nutrients from soil [20]. The spatial configuration of root systems, particularly their vertical distribution and biomass allocation within soil profiles, is significantly correlated with the water and nitrogen uptake efficiency [21,22]. Research has demonstrated that an optimal root architecture can increase the NUE while mitigating NO_3_^−^-N leaching losses, thereby reducing the risk of environmental contamination [23]. Irrigation management substantially influences root development patterns. In conventional flood irrigation practices, over 85% of the winter wheat root biomass is typically concentrated within the upper 40 cm of the soil layer [21,24,25,26,27,28,29], substantially limiting access to subsoil resources (e.g., water and nitrogen). Therefore, improving the spatiotemporal coordination among water, nitrogen, and root in the soil profile is an effective path to increasing crop productivity and resource use efficiency.

In this study, we hypothesized that, under water- and fertilizer-saving management, the implementation of MSF could delay leaf senescence at the grain-filling stage; increase root–water–nitrogen spatiotemporal coordination through reducing redundant tillering; facilitate the N transfer from straw to grains; and, ultimately, increase the GY and NUE levels. The specific objectives of this study were (1) to establish optimized nitrogen application strategies for achieving yield stability and nutrient efficiency in MSF systems, and (2) to elucidate the regulatory effects of MSF with different nitrogen levels on the physiological characteristics of winter wheat in the NCP.

## 2. Results

### 2.1. Dry Matter Accumulation (DMA)

**Figure 1:**
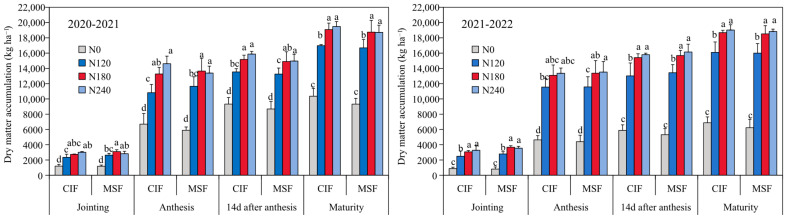
The effects of different irrigation modes with four nitrogen application levels on dry matter accumulation of winter wheat at different growth stage in 2020–2021 and 2021–2022 growing seasons. CIF and MSF represent conventional irrigation and fertilization and micro-sprinkling fertigation, respectively. N0, N120, N180, and N240 represent the nitrogen application rate of 0, 120, 180, and 240 kg ha^−1^. The different lowercase letters on the columns of the same year and the same growth period represent significant difference at *p* < 0.05.

The nitrogen application rate significantly influenced dry matter accumulation (DMA) in winter wheat (Figure 1). However, the irrigation method did not significantly affect DMA at the same nitrogen level. As shown in Figure 1, the progressive increase in nitrogen application from N0 to N180 significantly enhanced DMA at each growth stage under both conventional irrigation and fertilization (CIF) and MSF systems. Notably, no statistically significant differences in DMA were obtained between the N240 and N180 treatments (*p* > 0.05) under either irrigation method. These findings suggested that nitrogen application at a rate of 180 kg N ha^−1^ was reasonable for achieving a relatively high DMA and moderately reduced nitrogen input levels.

### 2.2. The Relative Leaf Chlorophyll Content (RLC)

**Figure 2:**
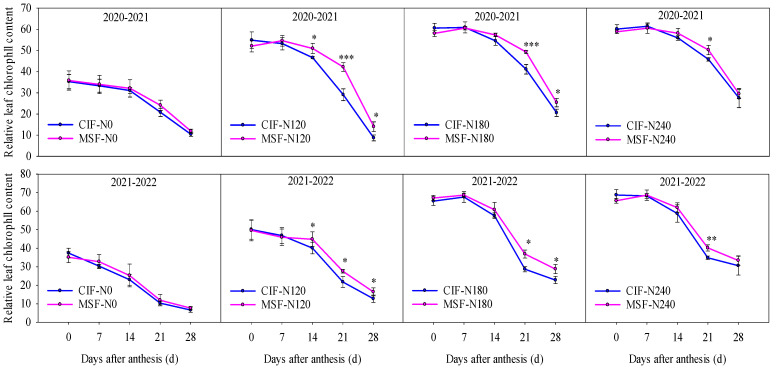
The effects of different irrigation modes with four nitrogen application levels on relative leaf chlorophyll content of winter wheat at different growth stage in 2020–2021 and 2021–2022 growing seasons. CIF and MSF represent conventional irrigation and fertilization and micro-sprinkling fertigation, respectively. N0, N120, N180, and N240 represent the nitrogen application rate of 0, 120, 180, and 240 kg ha^−1^. *, **, and *** indicate significant differences between CIF and MSF treatments under the same nitrogen fertilizer level in *t*-test at 0.05, 0.01, and 0.001 levels, respectively.

The RLC value progressively increased with an increasing nitrogen application rate under the same irrigation method (Figure 2). No significant differences (*p* > 0.05) in postanthesis RLC values were observed between the CIF-N0 and MSF-N0 treatments. However, MSF significantly increased the RLC value from 14 to 28 d after anthesis at relatively high nitrogen application levels (N120, N180, and N240). Specifically, compared with those under the CIF-N120 treatment, the MSF-N120 treatment exhibited increases of 9.71%, 29.34%, and 27.50% in the RLC values at 14, 21, and 28 d after anthesis, respectively (two-year average). Compared with those under the CIF-N180 treatment, the MSF-N180 treatment resulted in substantial improvements of 24.23% and 24.72% (two-year average) at 21 and 28 d after anthesis, respectively. Similarly, compared with that under the CIF-N240 treatment, the MSF-N240 treatment provided a 12.49% increase (two-year average) in the RLC value at 21 d after anthesis. These findings collectively indicated that the implementation of MSF effectively increased the RLC value at critical postanthesis developmental stages when combined with moderate nitrogen supplementation.

### 2.3. Net Photosynthetic Rate (NPR)

**Figure 3:**
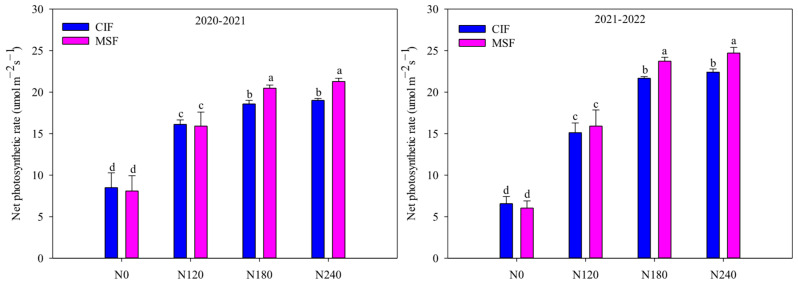
The effects of different irrigation modes with four nitrogen application levels on net photosynthetic rate of winter wheat at 14 d after anthesis in 2020–2021 and 2021–2022 growing seasons. CIF and MSF represent conventional irrigation and fertilization and micro-sprinkling fertigation, respectively. N0, N120, N180, and N240 represent the nitrogen application rate of 0, 120, 180, and 240 kg ha^−1^. The different lowercase letters on the columns of the same year represent significant difference at *p* < 0.05.

As shown in Figure 3, no significant differences in the net photosynthetic rate (NPR) were observed between CIF and MSF under either the N0 or N120 treatment. However, compared with CIF, MSF significantly increased the NPR at elevated nitrogen application levels, with increases of 9.85% and 11.11% (two-year average) under the N180 and N240 treatments, respectively. Notably, the NPR did not significantly differ between the N180 and N240 treatments under the same irrigation method. These findings suggested that adopting MSF combined with moderate nitrogen application (MSF-N180) effectively enhanced the photosynthetic material production capacity at the critical grain-filling stage of winter wheat.

### 2.4. Grain Weight Growth Dynamics

**Figure 4:**
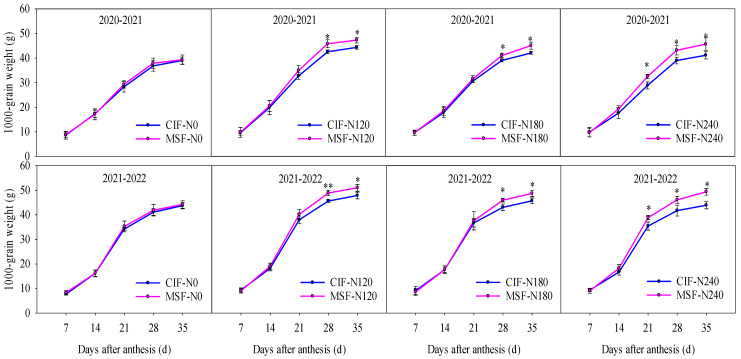
The effects of different irrigation modes with four nitrogen application levels on the grain weight growth dynamic at 7 d to 35 d after anthesis in 2020–2021 and 2021–2022 growing seasons. CIF and MSF represent conventional irrigation and fertilization and micro-sprinkling fertigation, respectively. N0, N120, N180, and N240 represent the nitrogen application rate of 0, 120, 180, and 240 kg ha^−1^. * and ** indicate significant differences between CIF and MSF treatments under the same nitrogen fertilizer level in *t*-test at 0.05 and 0.01 levels, respectively.

The evolution of the thousand-grain weight (TGW) during grain filling exhibited an S-shaped growth curve (Figure 4). Throughout the observation period, the TGW values did not significantly differ (*p* > 0.05) between the MSF-N0 and CIF-N0 treatments. However, under increasing nitrogen application, a distinct temporal widening gap emerged between the MSF and CIF treatments. While the initial grain-filling stages demonstrated comparable TGW values across all nitrogen levels, significant divergence was observed during the mid-to-late developmental phases. Compared with those under the CIF-N120, CIF-N180, and CIF-N240 treatments, the TGW under the MSF treatment increased by 6.23%, 2.64%, and 11.15%, respectively, at 21 d after anthesis; by 7.38%, 4.73%, and 10.50%, respectively, at 28 d after anthesis; and by 6.63%, 6.83%, and 11.74%, respectively, at 35 d after anthesis (two-year average). This differential growth pattern highlights the superior late-phase grain-filling capacity of MSF, which is beneficial for increasing the grain weight.

### 2.5. The Spatial Proportion of Root–Water–Nitrogen in Soil

**Figure 5:**
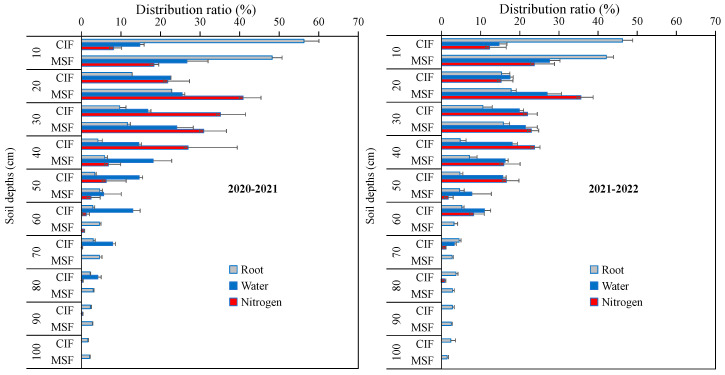
The effects of different irrigation modes under N180 on the distribution of water, nitrogen, and root spatial proportions in the 0–100 cm soil during the two growing seasons of 2020–2021 and 2021–2022. The “nitrogen” in this part was labeled with isotopes (^15^N). CIF and MSF represent conventional irrigation and fertilization and micro-sprinkling fertigation, respectively. N180 represent the nitrogen application rate of 180 kg ha^−1^.

Under the CIF treatment, the roots were mainly distributed in the 0–10 cm soil layer, accounting for an average of 51.2% of the total number of roots in the 0–100 cm layer over the two-year period (Figure 5). With the increasing depth of the soil layer, the root distribution ratio significantly decreased to 14.1% and 10.1% in the 10–20 cm and 20–30 cm soil layers, respectively, over the two years, and the roots in all the other soil layers (30–100 cm) accounted for less than 10% of the total roots. Compared with the CIF treatment, the MSF treatment significantly altered the root distribution ratio in the 0–100 cm soil layer; namely, the root distribution ratio decreased to 45.2% in the 0–10 cm soil layer but increased to 20.3% and 13.7% in the 10–20 cm and 20–30 cm soil layers, respectively. In the other soil layers (30–100 cm), the proportion of roots was less than 10%.

The distribution ratio of irrigation water in each soil layer differed between CIF and MSF (Figure 5). The water distribution ratio under CIF was 14.74% (two-year average) in the 0–10 cm soil layer. However, that under MSF reached as high as 27.13% in the 0–10 cm soil layer over the two years. Moreover, the water contribution ratios in the 10–20 cm and 20–30 cm soil layers under the MSF treatment were higher than those under the CIF treatment by 33.71% and 25.39%, respectively. This finding indicated that the soil layers with a more abundant root distribution under the MSF treatment exhibited relatively high moisture contents.

The distribution ratios of ^15^N under CIF in each soil layer also differed from those under MSF (Figure 5). The applied ^15^N was mainly concentrated in the 10–40 cm soil layer under the CIF treatment. Over the two years, the ^15^N concentration ratios in the 10–20 cm, 20–30 cm, and 30–40 cm soil layers were 18.52%, 28.57%, and 25.37%, respectively, whereas that in the 0–10 cm soil layer (roots are mainly distributed in this soil layer) reached only 10.21% under CIF. However, the soil ^15^N concentration ratio was primarily concentrated in the 0–30 cm soil layers under the MSF treatment. Over the same period, the average soil ^15^N concentration ratios were 21.04%, 38.26%, and 26.93% in the 0–10 cm, 10–20 cm, and 20–30 cm soil layers, respectively.

The above results indicated that there was a correspondingly greater distribution ratio of irrigation water and nitrogen in the soil layers, with a greater root distribution ratio under the MSF treatment than under the CIF treatment. This suggested a superior coupling of the water–nitrogen–root distributions under the MSF treatment.

### 2.6. Distribution of Nitrate Nitrogen in Soil at the Maturity Period

**Figure 6:**
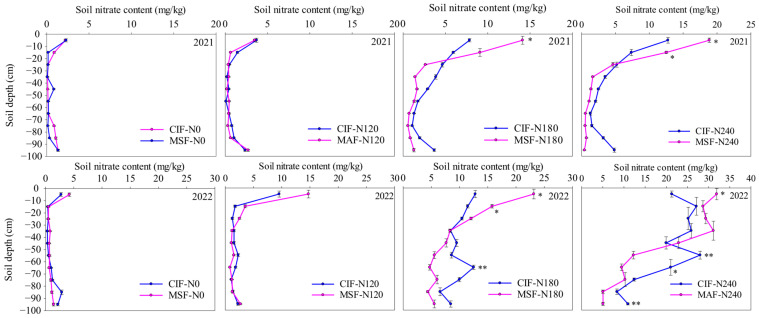
The effects of different irrigation modes with four nitrogen application levels on the distribution of soil nitrate nitrogen at maturity in 2020–2021 and 2021–2022. CIF and MSF represent conventional irrigation and fertilization and micro-sprinkling fertigation, respectively. N0, N120, N180, and N240 represent the nitrogen application rate of 0, 120, 180, and 240 kg ha^−1^. * and ** indicate significant differences between CIF and MSF treatments under the same nitrogen fertilizer level in *t*-test at 0.05 and 0.01 levels, respectively.

The soil nitrate nitrogen content (SNC) increased with the amount of nitrogen applied (Figure 6). No significant differences in the SNC were obtained between the CIF and MSF treatments at equivalent nitrogen application rates (N0 and N120). However, compared with the SNC under CIF-N180, that under MSF-N180 significantly increased by 55.63% and 81.11% in the 0–10 cm soil layer between 2020–2021 and 2021–2022, respectively, and increased by 37.67% in the 10–20 cm soil layer between 2021–2022. The SNC under the MSF-N180 treatment in the 60–70 cm soil layer was significantly lower than that under the CIF-N180 treatment by 62.21% between 2021–2022. Compared with the SNC under CIF-240, that under MSF-N240 significantly increased by 48.86% in the 0–10 cm soil layer and 70.06% in the 10–20 cm soil layer (two-year average). Nevertheless, compared with that under CIF-240, MSF-N240 yielded a lower SNC in deep soil. Between 2020–2021, compared with those under CIF-240, the SNC in the 80–90 cm and 90–100 cm soil layers significantly decreased by 77.34% and 91.17%, respectively, under MSF-N240. Moreover, the SNC in the 50–60 cm, 60–70 cm, and 90–100 cm soil layers significantly decreased by 56.23%, 55.03%, and 53.75%, respectively, under MSF-N240. This indicated that, under medium- and high-nitrogen fertilizer application rates (180–240 kg ha^−1^), MSF could maintain a relatively high SNC in the topsoil (0–30 cm) while reducing the leaching of nitrate nitrogen to deeper soil layers (Figure 6).

### 2.7. Population and Yield Characteristics at Maturity

Irrigation methods with different nitrogen application rates significantly affect the population characteristics (Table 1). Although, compared with those under CIF, MSF reduced ITN, ITW, and EN_-10_ under both the N0 and N120 treatments, the differences were not statistically significant. However, under relatively high nitrogen application rates (N180 and N240), MSF significantly reduced these parameters. Specifically, under the N180 treatment, MSF decreased the ITN, ITW, and EN_-10_ by 24.18%, 36.36%, and 26.47%, respectively. Greater reductions of 50.99%, 49.20%, and 38.28%, respectively, were observed under the N240 treatment (two-year averages compared with those under CIF). Under both the CIF and MSF treatments, the ITN, ITW, and EN_-10_ progressively increased with an increasing nitrogen application rate. Notably, under CIF, the N240 treatment resulted in significantly greater values for these parameters than those under the treatments with the other nitrogen application rates (N0–N180). In contrast, MSF resulted in no statistically significant differences in the ITN, ITW, or EN_-10_ between the N180 and N240 treatments.

The irrigation methods significantly influenced yield components under the different nitrogen application rates, as detailed below (Table 1). Compared with CIF, MSF reduced the EN, with statistically significant differences observed under N240 between 2020–2021 and under both N180 and N240 between 2021–2022. Conversely, MSF enhanced the number of grains (GN) across all nitrogen treatments except N0, particularly demonstrating a significant superiority over CIF under the N180 treatment. The TGW values were consistently greater under MSF than under CIF across all nitrogen levels, reaching statistical significance under N120 and N240. Under the same irrigation method, the EN value increased with an increasing nitrogen application rate. However, the maximum GN value was reached under N180, and the maximum TGW value was attained under N120.

Compared with CIF, MSF consistently provided significantly greater GY and HI values under both N180 and N240 (Table 1). The analysis revealed that the MSF-N180 and MSF-N240 treatments increased the GY by 9.09% and 9.72%, respectively, while increasing the HI by 9.72% and 8.86%, respectively, compared with those under CIF for the same nitrogen application rate. Furthermore, nitrogen application exerted a dose-dependent effect on yield enhancement across both irrigation methods, although no statistically significant differences (*p* > 0.05) were obtained between the N180 and N240 treatments for the same irrigation method. Notably, even at the lower nitrogen level (N120), MSF maintained a significant 7.58% increase in the GY relative to CIF during the 2021–2022 growing season, highlighting its superior yield-improving effects.

According to the above results, under medium nitrogen (N180) levels, MSF could reduce the ITN, increase the GN and TGW, increase the HI, and, ultimately, enhance the GY compared with CIF.

### 2.8. The Pearson Correlation Analysis

**Figure 7:**
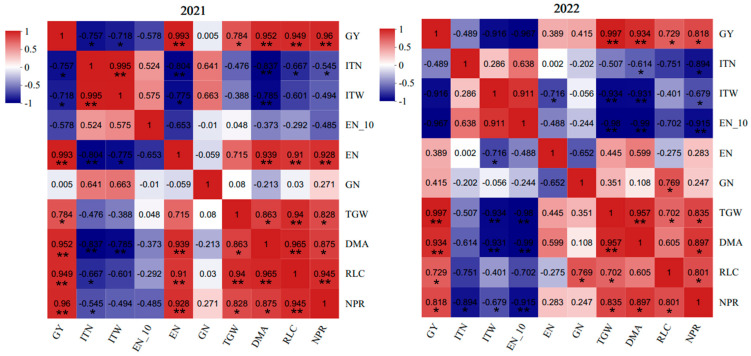
Pearson correlation analysis among indicators related to grain yield. ITN, inefficient tiller number; ITW, dry weight of the inefficient tillers; EN_−10_, number of ears with less than 10 grains; EN, ear number; GN, grain number; TGW, 1000−grain weight; GY, grain yield; DMA, dry matter accumulation; RLC, relative leaf chlorophyll content; and NPR, net photosynthetic rate. * and ** represent significant difference at *p* < 0.05 and *p* < 0.01, respectively.

Figure 7 presented the results of a Pearson correlation analysis among grain yield (GY)-related traits (Figure 7). GY exhibited significantly positive correlations with thousand-grain weight (TGW), dry matter accumulation (DMA), relative leaf chlorophyll content (RLC), and net photosynthetic rate (NPR). Conversely, GY showed remarkably negative correlations with ineffective tiller number (ITN) and ineffective tiller weight (ITW), and negative correlations with EN_-10_. TGW exhibited significantly positive correlations with RLC, NPR, and DMA. Furthermore, significantly positive correlations were observed between DMA and both RLC and NPR, as well as between NPR and RLC. These results suggested that enhancing the RLC and reducing tiller redundancy through improved irrigation and fertilization contributes to increased NPR, thus improving DMA, TGW, and then GY.

### 2.9. Characteristics of Nitrogen Accumulation and Distribution

The irrigation method and nitrogen application rate significantly influenced nitrogen accumulation and distribution (Table 2). Compared with CIF, MSF decreased the NA, NITM, and NSM at the same nitrogen rate during the two growing seasons. The differences in the NA, NITM, and NSM between MSF and CIF reached significant levels under N180 and N240. Compared with CIF, MSF significantly increased the NGM under N240; NPA under N180 and N240; and CRPATG under N120, N180, and N240. However, there was no significant difference in the NTM between CIF and MSF. Compared with that under CIF, the nitrogen harvest index (NHI) under MSF at N120, N180, and N240 significantly increased by 3.83%, 5.13%, and 7.83%, respectively, between 2020–2021 and by 3.61%, 4.55%, and 6.72%, respectively, between 2021–2022. With an increasing nitrogen application rate, NA, NITM, NSM, NGM, NTM, NPA, and CRPATG all increased under CIF and MSF. However, the NHI showed the opposite trend.

In summary, compared with CIF, MSF effectively increased the N accumulation in grains and post anthesis plants, decreased the N accumulation in inefficient tillers and plant straw, and then reduced the N redundancy and facilitated N allocation optimization.

### 2.10. Nitrogen Use Efficiency (NUE)

The irrigation method and nitrogen application rate significantly affected the NUE (Table 3). Compared with those under CIF, the N partial factor productivity (NPFP), N agronomic efficiency (NAE), and N physiological efficiency (NPE) significantly increased by 8.97%, 22.13%, and 19.55% (two-year average), respectively, under N180 and N240. The NIUE under MSF was slightly greater than that under CIF at the same nitrogen application rate. The NPFP, NAE, NIUE, and NPE decreased with an increasing nitrogen application rate. These findings indicated that the MSF treatment increased the NPFP, NAE, and NPE under medium (N180) and high (N240) nitrogen application rates.

**Figure 8:**
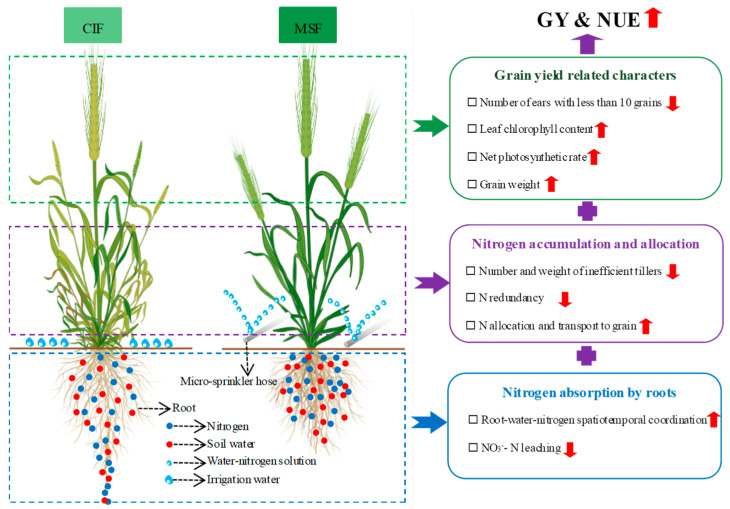
Schematic illustration of MSF simultaneously increasing grain yield and nitrogen use efficiency. CIF and MSF represent conventional irrigation and fertilization and micro-sprinkling fertigation, respectively.

In sum, the physiological mechanism for simultaneously increasing the GY and NUE under MSF (frequent small-dose irrigation and fertilization, N180) could be summarized as follows: it enhanced the grain-filling capacity, optimized C and N accumulation and allocation, and improved the spatiotemporal configuration of root–water–nitrogen (Figure 8).

## 3. Discussion

### 3.1. Increased Matter Productivity During Grain-Filling Stage Under MSF with Moderate Nitrogen Application

DMA after anthesis is a key characteristic for increasing GY levels [30]. Increasing the RLC of functional leaves can increase the photosynthetic capacity of the leaves and thereby enhance the accumulation of photosynthetic products. Reasonable water and fertilizer management can improve the biomass accumulation and distribution in plants [31]. MSF, where fertilizer is dissolved in water for precise irrigation and fertilization at the key growth stages of crops, which is an innovative irrigation technique that has emerged in recent years, merges the benefits of both drip and sprinkler irrigation systems [32,33]. Previous studies have revealed that applying water and fertilizer at low doses and high frequencies could delay flag leaf senescence, promote DMA during the filling period, and increase the GY [34,35]. In this two-year study, compared with the CIF treatment, the MSF treatment increased the relative leaf chlorophyll content at the entire grain-filling stage (7–28 d after anthesis) (Figure 3) and increased the NPR at 14 d after anthesis (Figure 4) by reducing the application of water and fertilizer before anthesis while increasing it during the grain-filling stage. Studies have suggested that micro-sprinkling irrigation enhances the accumulation of dry matter after flowering [35,36]. However, in this study, it should be noted that DMA under MSF did not significantly differ from that under CIF during each period. Furthermore, we isolated ineffective tillers from the entire plant and determined that the ITN and ITW under MSF were significantly greater than those under CIF (Table 2). This can also be regarded as MSF increasing mainly DMA in the tillering and stem parts that form spikes.

Nitrogen is a necessary nutrient for plant growth and development. Allocating more nitrogen to late application increased the crop yield through optimizing the DMA and dry matter partitioning and was an efficient nitrogen fertilizer management strategy under nitrogen reduction and fertigation [37]. Moderate nitrogen reduction increased the proportion of dry matter allocated to leaf translocation from vegetative to reproductive organs [38]. Additionally, in this study, the RLC, NPR, and DMA increased with an increasing nitrogen application rate under CIF or MSF, whereas those characteristics did not significantly differ between N180 and N240. These results suggested that adopting MSF with a moderate nitrogen application rate (180 kg ha^−1^) could supplement nitrogen at the anthesis and filling stages and alleviate premature leaf senescence caused by nitrogen deficiency. This process helps maintain the greater photosynthetic and physiological capabilities at the middle-to-late grain-filling stages and ultimately increases the grain weight and yield.

### 3.2. Optimized N Accumulation and Allocation, and Increased Grain Yield (GY) Under MSF with Moderate Nitrogen Application

The ideotype population is the basis for high-yield and efficient wheat cultivation, which is evaluated on the basis of population uniformity [39]. The wheat plant architecture, comprising main stems and tillers [25,40], demonstrates that tiller fertility influences the final GY during winter wheat cultivation [30,41]. Optimized nitrogen application and irrigation reduced the number of ineffective tillers during the flowering period, increased the number of spikes, and enhanced the uniformity of the population [39]. However, excessive nitrogen inputs paradoxically increased the total numbers of both tillers and ineffective tillers, thus exacerbating intraplant competition and ultimately reducing the GY [42]. Research has confirmed that a relatively high nitrogen application ratio at sowing or during the vegetative period stimulates excessive leaf growth and promotes the production of nonproductive tillers [39,43]. Conversely, low nitrogen (LN) inhibited the tiller bud growth and reduced the tiller occurrence by decreasing the sucrose content within the buds. Furthermore, LN promoted primary and lateral root growth, thereby competing with the tiller buds for nitrogen and sucrose [44]. Additionally, the lower soil moisture during the early stage increased the ABA concentrations in the inferior tillers, inhibiting their growth [45]. Similar results were obtained in this study, which revealed that irrigation methods and nitrogen regimes significantly affect the number of ineffective tillers, nitrogen allocation patterns, and, ultimately, GY. MSF employed in this study demonstrated precise control over the irrigation volume, fertilization timing, and nutrient delivery. Compared with CIF, MSF reduces the supply of water and fertilizer at the early stage, prevents excessive population growth, and optimizes the population structure, which is manifested mainly in the reduction in the number of ineffective tillers and the occurrence of low-yield ears (<10 grains/ear, EN_-10_).

Increasing the nitrogen application rate increased the nitrogen absorption levels [46]. In this study, CIF promoted redundant nitrogen uptake by inefficient tillers, increased the nitrogen accumulation in both ineffective tillers and mature straw, and decreased the amount of nitrogen accumulation in grains at maturity. In contrast, MSF implementation effectively mitigated nitrogen redundancy through optimized uptake and allocation, thereby increasing the NHI. Previous studies indicated that optimizing the base-to-topdressing ratio of nitrogen fertilizer (5:5) under micro-sprinkling irrigation significantly enhanced the nitrogen metabolism and ultimately increased the NUE [34], which is similar to the findings of this study. In this study, increasing the fertigation frequency at anthesis and implementing MSF increased the nitrogen absorption pathway of plants through leaves, which was affected by the foliar fertilizer and enhanced nitrogen absorption. Improving irrigation methods and optimizing nitrogen fertilizer management can significantly increase yields. Notably, under medium (N180)- and high (N240)-nitrogen regimes, MSF outperformed CIF in terms of yield production by 8.57% (two-year average), which was achieved mainly by increasing the number of grains (GN) and TGW, demonstrating its potential for sustainable yield intensification in winter wheat cultivation.

### 3.3. Enhanced Nitrogen Use Efficiency Through High Root–Water–Nitrogen Coupling in Soil Under MSF

Nitrate nitrogen (NO_3_^−^-N) serves as the primary nitrogen source absorbed by plant roots from soil, and its distribution is modulated by irrigation practices and fertilization. Excessive irrigation and nitrogen application can induce NO_3_^−^-N leaching into deeper soil layers [17]. In this study, CIF resulted in significantly greater NO_3_^−^-N accumulation in deep soil layers (>50 cm) at maturity than MSF did under medium (N180) and high nitrogen application rates (N240), which constrained the increase in the NUE. This can be attributed to two interrelated factors. First, the frequent but low-volume irrigation characteristic of MSF minimized the nitrogen leaching losses caused by excessive water percolation. Second, the split application of nitrogen fertilizers through fertigation ensured a steady supply of NO_3_^−^-N at critical growth stages, which matches the dynamic nutrient demands of crops. Previous studies have also shown that increasing the ratio of the base fertilizer significantly increased the content of NO_3_^−^-N residue in deep soil, and NO_3_^−^-N was distributed mainly in the 0–40 cm soil layer with an increasing topdressing ratio [34]. Similar results were also obtained in this study. MSF maintained adequate nitrogen availability in the upper soil layers (0–30 cm), thereby increasing the nitrogen absorption efficiency (Figure 5). This contrasts sharply with CIF, in which low-frequency and high-dose nitrogen application often exceeds plant requirements. This finding suitably agrees with the results of Chen et al. [47], who reported that soil NO_3_^−^-N was transported with soil water, and, with an increasing fertilization amount, the NO_3_^−^-N concentration increased accordingly. The fertilization amount and strategy thus influenced the spatial distribution pattern of soil N and the risk of nitrate leaching.

The water and nitrogen contents in the main distribution soil layer of crop roots were adequate, which is conducive to the efficient utilization of resources by crops [12]. Compared with the CIF treatment, the MSF treatment significantly altered the root distribution of winter wheat, resulting in an increase in the root distribution in the upper soil layers (0–30 cm) and a decrease in the root distribution in the deeper soil (Figure 5). Previous research has shown that root growth was most stimulated in the topsoil layers but inhibited in deeper soil under high-frequency sprinkler irrigation [48]. In this study, under the MSF treatment, the moisture and nitrogen contents in the upper soil layer were relatively high through increasing irrigation frequencies, indicating a favorable coupling of the water, nutrient, and root systems. Moreover, the MSF treatment significantly increased the NUE, including the NPFP, NAE, and NPE, especially under medium (N180) and high nitrogen fertilizer inputs (N240). This precision enhanced the synchronized uptake of water and nutrients, subsequently increasing the NUE. Moreover, the irrigation water use efficiency (GY/total irrigation amount) was significantly improved by 9.1% under MSF compared to CIF. These findings establish MSF (small and frequent) as an environmentally sustainable and agronomically efficient water and fertilizer management strategy that effectively balances increased productivity with ecological protection. Although this study elucidated the regulatory effects of MSF on wheat yield and nitrogen use efficiency through a series of physiological indicators, the molecular level mechanisms, such as the gene expression involved in photosynthesis, hormone signaling associated with leaf senescence, and soil microbial communities, warrant further research. These will be the focus of our subsequent work.

## 4. Materials and Methods

### 4.1. Experimental Site

**Figure 9:**
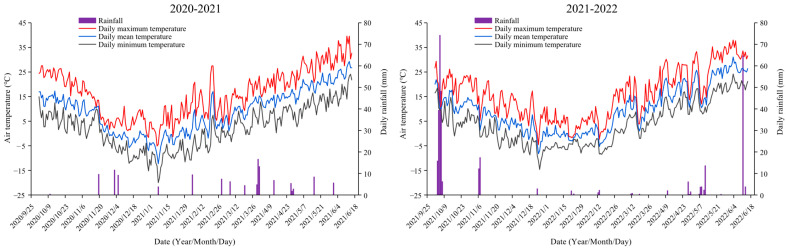
Daily maximum temperature, minimum temperature, mean temperature, and rainfall during the 2020–2022 growing season in winter wheat.

Field experiments were conducted during 2020–2021 and 2021–2022 growing seasons at the Dishang Experimental Station (114.73° E, 37.95° N; altitude 21.0 m) of the Hebei Academy of Agricultural and Forestry Sciences in Hebei Province, which represents the climate conditions and prevailing agricultural production of NCP. The experimental site experiences a frost-free period of 188 d. The annual evaporation was 510 mm, and the average temperature is 12.8 °C. The soil type at the research site was a typical silt loam soil consisting of 4.46% clay, 75.56% silt, and 19.98% sand. At the beginning of the experiment, organic matter, total nitrogen, available P, available K, and pH in the topsoil (0–20 cm) were 15.6 g kg^−1^, 1.0 g kg^−1^, 32.1 mg kg^−1^, 107.2 mg kg^−1^, and 8.0, respectively (determined by Institute of Soil Science, Chinese Academy of Sciences, Nanjing, China). The total precipitation was 120.5 mm and 199.6 mm during the winter wheat growing season in 2020–2021 and 2021–2022, respectively. Figure 9 shows the monthly rainfall and daily mean air temperature during the two years.

### 4.2. Experimental Design

#### 4.2.1. Field Experiment

A widely used winter wheat variety in the NCP, Jimai 325 (JM325), which performed exceptionally well in characteristics of water saving, was chosen as the experimental material. The experiment was carried out in a split-plot design. In the main plots, two irrigation methods were applied during the growing seasons, namely, conventional irrigation and fertilization (CIF) and micro-sprinkling fertigation (MSF). CIF was conducted using a polyvinyl chloride (PVC) pipe with a diameter of 20 cm. MSF utilized a micro-sprinkling hose measuring 10 m in length and 32 mm in width. Each hose contained 50 orifice groups spaced at 20 cm intervals. Each group comprised five orifices, all with a diameter of 0.8 mm. The hose wall thickness was 0.2 mm. Each experimental plot was equipped with four such hoses spaced 1.8 m apart. The hose had a flow rate of 1.0 m^3^ h^−1^ at an operating pressure of 0.1 MPa. Irrigation volume was controlled by an electronic flow meter installed at the head of the micro-sprinkling irrigation system. Venturi injector, a simple fertilizing device which utilized pressure difference to transport liquid fertilizer to the fields, was used in the MSF system. Four N application rates (0, 120, 180, and 240 kg ha^−1^, noted by N0, N120, N180, and N240) were assigned in subplots. Each treatment had three replications in 2020–2021 and four replications in 2021–2022, with an area of 72 m^2^ (10 m × 7.2 m) for each plot, and each experimental plot consisted of 48 rows of wheat spaced 15 cm apart. The planting density was 4.5 × 10^6^ seeds ha^−1^. Before sowing, a certain percentage of N based on different treatments, 120 P_2_O_5_ kg ha^−1^ and 90 K_2_O kg ha^−1^, were applied as basal fertilizer. The urea, superphosphate, and potassium chloride were the sources of N, P_2_O_5_, and K_2_O, respectively. The irrigation time, irrigation amount, and nitrogen application ratio for each treatment were shown in Table 4. For CIF, the remaining portions of nitrogen were manually applied before irrigation. For MSF, the remaining portions of N as topdressing were completely dissolved in a fertilization device and applied together with the irrigation water. Irrigation water use efficiency was calculated by dividing grain yield by the amount of irrigation water applied. The winter wheat was sown on 16 October 2020 and 25 October 2021, and harvested on 6 June 2021 and 11 June 2022, respectively. Weed, disease, and pest management were referred to general field management practices.

#### 4.2.2. Micro-Plot Experiment

At the same growing seasons, a micro-plot was set in the middle of field experiment site for ^15^N isotope marking. In the micro-plot, the CIF mode was simulated by slowly and evenly applying water to the wheat roots using a measuring cup. The MSF mode was mimicked using an agricultural sprayer to uniformly spray the water and fertilizer solution. The total N application amount in micro-plot was 180 kg ha^−1^. The basal application of common urea (50%) + topdressing application of ^15^N labeled urea (50%) was set under the MSF to determine the spatial allocation of nitrogen, water, and root in the soil. ^15^N-labeled urea with 10.24% abundance and 46.83% nitrogen concentration was produced by Shanghai Research Institute of Chemical Industry. The micro-plot area was 0.45 m^2^, with 5 rows of wheat. Zn-coated iron frames with dimensions of 0.75 m × 0.6 m × 0.4 m (length × width × height) were used to separate the micro-plot from field, and the iron frames were 5 cm above the ground to prevent ^15^N loss and external nitrogen contamination. Following calculation, 8.6 g of common urea was applied as basal fertilizer to each micro-plot, and 8.6 g of isotopically labeled urea (containing 0.88 g of ^15^N) was applied as topdressing fertilizer at the jointing stage. Three repetitions were established for each micro-plot treatment. Other weed and pest management was the same as field management.

### 4.3. Sampling and Measurements

#### 4.3.1. Dry Matter Accumulation

All plants at ground level of two rows with 0.5 m length (avoiding border rows) in each plot were sampled at jointing, anthesis, 14 d after anthesis, and maturity, and were oven-dried at 75 °C to constant weight to determine above-ground dry matter accumulation at different growing stages.

#### 4.3.2. Population Traits, Yield Characteristics, and Harvest Index at Maturity

At maturity, the total number of ears (EN), the number of ears with fewer than 10 grains (EN_-10_), and the number of ineffective tillers that have not formed spikes (ITN) were counted in a representative sampling area (two rows with 1 m, 0.3 m^2^). Following this, 20 wheat plants were selected randomly, and the average grain number per spike (GN) was calculated, after which the samples were divided into three parts: ineffective tillers that have not formed spikes, grains, and residual straw. Each component was then dried to a constant weight at 75 °C to record the dry matter (DM). The total above-ground dry matter accumulation (TDM) was the sum of DM of each part. The 1000-grain weight (TGW) was calculated by weighing 1000 grains. In each plot, all spikes of a representative 4 m^2^ were harvested and threshed for grain yield (GY) calculation. GY was converted to a 13% standard moisture. The harvest index was calculated as the ratio of GY to TDM.

#### 4.3.3. Grain Weight Dynamics

At anthesis, the representative spikes with similar growth and flowering at the same day were tagged with labels in each plot. Sampling starts at 7th day after anthesis and the sampling interval was 7 days until harvest. Ten individual spikes were taken each time. All spikes sampled each time were dried at 75 °C to constant weight, and the thousand-grain weight was measured to analyze the grain weight dynamics at filling stage.

#### 4.3.4. The Relative Chlorophyll Content and Photosynthetic Rate

At anthesis, and 7th, 14th, 21st, and 28th day after anthesis, a portable SPAD-502 meter (Konica Minolta, Tokyo, Japan) was employed to measure the SPAD values of the flag leaf in each plot as indirect assessment of relative leaf chlorophyll content. Fifty representative flag leaves were selected in each plot. The photosynthetic rate of flag leaves was determined by LI-6400 portable photosynthesis measurement system (LI-COR, Lincoln, NE, USA). For each plot, 10 flag leaves with the same light direction and the same growth were selected under natural light in the field, and the photosynthetic rate of flag leaves was measured from 9:00 to 11:00 at 14th day after anthesis.

#### 4.3.5. The Spatial Coordination of Root–Water–Nitrogen in Soil

The spatial coupling characteristics of the water–nitrogen–root in CIF-N180 and MSF-N180 were examined at jointing stage which was the key growing stage of water and fertilizer absorption peak in wheat. The soil of each micro-plot was sampled from 0 to 100 cm at 10 cm intervals using a soil corer at 48 h after fertigation. One part of the sampled soil was used to determine the soil water content, and the other part is used to determine the ^15^N abundance. Soil gravimetric water content (SWC) was measured by oven-drying samples at 105 °C to a constant weight. ^15^N abundance of sampled soil was determined using stable isotope ratio mass spectrometry (IRMS). Meanwhile, the root under every soil layer was collected to analyze the difference in root distribution ratio under the two irrigation modes. Each sampled soil was placed in a mesh bag and we rinsed them until only the roots remain.

SWC (%) = (fresh soil weight − dry soil weight)/dry soil weight(1)

SWS (mm) = SWC × soil bulk density × 100(2)

WDR (%) = SWS/irrigation amount(3)

where SWC is the soil gravimetric water content; SWS is the soil water storage at every soil layer; and WDR is the distribution ratio of irrigation water on every soil layer.

SNA (kg ha^−1^) = 10^4^ × 0.1 × soil bulk density × ^15^N abundance/10^6^ (4)

NDR (%) = SNA/nitrogen application amount(5)

where SNA is the nitrogen accumulation in each layer of soil; 10^4^ is the area of per hectare, and 0.1 is the thickness of each layer of soil. NDR is the distribution ratio of nitrogen on every soil layer.

SDR (%) = root dry weight in each soil layer/total root dry weight in 0–100 cm(6)

where SDR is the distribution ratio of root on every soil layer.

#### 4.3.6. Soil NO_3_^−^-N Content and Plant Nitrogen Accumulation and Distribution

At anthesis, the soil in each treatment was sampled at 10 cm intervals between 0–100 cm to determine the soil NO_3_^−^-N contents, which were extracted with 0.01 mol L^−1^ CaCl_2_ and determined using an ultraviolet spectrophotometer (UV2600, Shimadzu, Japan). The calculation method for the accumulated amount of NO_3_^−^-N in 1 m soil is the sum of nitrate accumulation in each layer [18].

At anthesis and maturity, all representative plants of 0.3 m^2^ in each plot were sampled and divided into inefficient tiller, and straw without inefficient tiller and grain (only at maturity), and we dried them at 75 °C to constant weight. The nitrogen content in samples was determined using the Kjeldahl method [49]. The dry matter accumulation (DMA) and nitrogen content (N%) of each part were recorded separately.

NA (kg ha^−1^) = the sum of DMA of each part at anthesis × N% in each part (7)

NITM (kg ha^−1^) = the DMA of inefficient tiller at maturity × N% in inefficient tiller (8)

NSM/NGM/NTM (kg ha^−1^) = the DMA of straw/grain/total plant at maturity × N% in straw/grain/total plant (9)

NPA (kg ha^−1^) = NT−NA (10)

CRPATG (%) = NPA/NGM(11)

NHI (%) = NG/NT(12)

where NA is the total nitrogen accumulation at anthesis; NITM is the nitrogen accumulation of inefficient tiller; NSM/NGM/NTM represent the nitrogen accumulation of straw, grain, and total plant at maturity, respectively; NPA is the nitrogen accumulation from post-anthesis to maturity; and CRPATG is the post-anthesis nitrogen translation contribution to the grain N.

#### 4.3.7. Nitrogen Use Efficiency

In this study, four kinds of nitrogen use efficiency (NPFP, NAE, NIUE, and NPE) were used to clarify the difference among treatments from different perspectives. The calculation formulae are as follows [50].

NPFP (kg/kg) = GY/NAM(13)

NAE (kg/kg) = (GYNx − GYN0)/NAM(14)

NIUE (kg/kg) = GY/NU(15)

NPE (kg/kg) = (GYNx − GYN0)/(NUx − NU0)(16)

where NPFP is the nitrogen partial factor productivity; NAE is the nitrogen agronomic efficiency; NIUE is the nitrogen internal utilization efficiency; NPE is the nitrogen physiological efficiency; GY is the grain yield (kg ha^−1^); NAM is the nitrogen application amount during the whole growing season (kg ha^−1^); and N0 and Nx represent fertilizer N applied at a rate of 0 and x kg ha^−1^. In this study, x represents 120, 180, and 240. GYNx and GYN0 are the grain yields under Nx and N0, respectively. NU is crop N uptake (kg ha^−1^). NUx and NU0 represent crop N uptake measured in above-ground biomass (kg ha^−1^) that applied fertilizer N at a rate of x and 0 kg ha^−1^.

### 4.4. Statistical Analysis

Statistical analyses were conducted in SPSS 27.0 software (SPSS Inc., Chicago, IL, USA). An analysis of variance (ANOVA) is carried out to determine the statistical significance of differences between mean values. Multiple comparisons were conducted using the least significant difference (LSD) at the 5% probability level. All figures were plotted using SigmaPlot 14.0 (Systat Software, Inc., SanJose, CA, USA).

## 5. Conclusions

MSF significantly increased the GY and NUE of winter wheat under moderate nitrogen application (180 kg N ha^−1^). The GY improvement could be attributed primarily to the after effects of MSF through the optimized nitrogen absorption and allocation pattern. Additional irrigation and fertilization at the grain-filling stage effectively delayed leaf senescence, elevated leaf photosynthetic and grain-filling capacities, and promoted population structural optimization. Compared with CIF, the GN, TGW, and the final GY were significantly increased under MSF. Furthermore, MSF improved N accumulation and allocation patterns by reducing redundant N redundancy in nonproductive tillers while increasing root–water–nitrogen spatiotemporal coordination. This optimization decreased nitrogen leaching losses and increased the NUE compared with those under CIF. In summary, MSF can be recommended as a high-yield and efficient cultivation technique in the NCP.

## Figures and Tables

**Table 1:** Population and yield characteristics of different irrigation methods with four nitrogen application rates at maturity in 2020–2021 and 2021–2022.

Year	Irrigation Methods	Nitrogen Application Rates	ITN (×10^6^ ha^−1^)	ITW (kg ha^−1^)	EN_-10_ (×10^6^ ha^−1^)	EN (×10^6^ ha^−1^)	GN	TGW (g)	GY (kg ha^−1^)	HI (%)
2020–2021	CIF	N0	0.2 ± 0.1 d	55.7 ± 24.8 e	0.2 ± 0.0 e	3.0 ± 0.2 d	27.4 ± 1.6 d	43.7 ± 1.4 e	4915.5 ± 482.3 d	0.41 ± 0.001 de
		N120	1.0 ± 0.3 b	585.7 ± 61.0 cd	0.4 ± 0.1 c	4.5 ± 0.4 c	36.2 ± 1.1 c	47.9 ± 0.7 bc	8530.3 ± 292.5 c	0.44 ± 0.012 bc
		N180	1.1 ± 0.6 b	996.1 ± 126.7 b	0.5 ± 0.0 b	5.4 ± 0.3 ab	38.4 ± 1.9 bc	46.4 ± 0.5 cd	8992.4 ± 155.8 bc	0.41 ± 0.009 de
		N240	2.6 ± 0.3 a	1324.5 ± 154.2 a	0.7 ± 0.1 a	5.8 ± 0.3 a	38.4 ± 1.1 bc	43.9 ± 1.5 de	8892.7 ± 80.8 c	0.40 ± 0.008 e
	MSF	N0	0.3 ± 0.1 cd	93.7 ± 86.9 e	0.2 ± 0.0 e	3.2 ± 0.3 d	24.7 ± 3.1 d	44.2 ± 1.9 de	4582.3 ± 469.2 d	0.43 ± 0.016 cd
		N120	0.8 ± 0.3 bc	501.4 ± 80.5 d	0.2 ± 0.0 de	4.3 ± 0.5 c	37.2 ± 3.8 c	51.0 ± 1.2 a	8675.4 ± 328.6 c	0.45 ± 0.014 ab
		N180	1.0 ± 0.4 b	714.1 ± 78.0 c	0.3 ± 0.1 cd	5.1 ± 0.1 b	43.0 ± 1.3 a	48.5 ± 1.5 bc	9695.4 ± 462.5 a	0.46 ± 0.002 a
		N240	1.2 ± 0.4 b	711.3 ± 50.1 c	0.3 ± 0.1 cd	5.2 ± 0.2 b	41.2 ± 0.8 ab	48.9 ± 0.9 ab	9534.4 ± 150.7 ab	0.44 ± 0.008 ab
2021–2022	CIF	N0	0.3 ± 0.2 e	95.3 ± 9.7 e	0.3 ± 0.1 de	5.4 ± 0.7 d	26.8 ± 1.5 e	43.7 ± 1.2 d	3462.3 ± 360.4 d	0.44 ± 0.007 c
		N120	1.5 ± 0.2 cd	684.3 ± 56.9 c	0.3 ± 0.0 cde	6.4 ± 0.3 c	31.0 ± 1.6 d	47.9 ± 1.4 bc	8883.3 ± 346.1 c	0.47 ± 0.013 b
		N180	2.4 ± 0.2 b	974.0 ± 129.8 b	0.6 ± 0.1 b	7.5 ± 0.3 ab	35.4 ± 2.1 c	46.4 ± 2.3 cd	9881.0 ± 278.7 b	0.46 ± 0.01 b
		N240	3.3 ± 0.2 a	1182.8 ± 104.1 a	0.7 ± 0.1 a	8.1 ± 0.2 a	36.3 ± 0.5 bc	43.9 ± 1.5 d	9612.8 ± 194.5 b	0.46 ± 0.013 b
	MSF	N0	0.3 ± 0.0 e	100.3 ± 18.6 e	0.3 ± 0.0 e	5.4 ± 0.1 d	25.7 ± 2.9 e	44.2 ± 1.5 d	3134.4 ± 536.8 d	0.44 ± 0.005 c
		N120	1.2 ± 0.3 d	530.7 ± 128.3 d	0.3 ± 0.0 cde	6.1 ± 0.7 c	40.0 ± 0.7 a	51.0 ± 1.4 a	9557.1 ± 266.2 b	0.50 ± 0.011 a
		N180	1.4 ± 0.4 d	734.0 ± 98.5 c	0.4 ± 0.1 cd	6.3 ± 0.2 c	39.4 ± 0.9 a	48.5 ± 1.7 bc	10904.3 ± 267.7 a	0.50 ± 0.009 a
		N240	1.7 ± 0.2 c	824.9 ± 74.2 c	0.4 ± 0.0 c	7.2 ± 0.2 b	38.6 ± 1.0 ab	49.4 ± 1.3 ab	10465.5 ± 235.6 a	0.49 ± 0.005 a
ANOVA (F-value)	Y		38.1 ***	1.0 ns	19.3 ***	434.8 ***	10.5 **	97.4 ***	9.1 **	228.7 ***
	I		48.9 ***	76.2 ***	115.2 ***	25.2 ***	21.6 ***	47.7 ***	23.0 ***	102.4 ***
	N		98.5***	301.6 ***	80.4 ***	114.8 ***	134.2 ***	39.7 ***	924.2 ***	35.2 ***
	Y × I		3.7 ns	1.6 ns	1.6 ns	5.4*	3.8 ns	0.0 ns	2.7 ns	1.2 ns
	Y × N		3.8 *	0.9 ns	1.6 ns	2.5 ns	2.1 ns	0.8 ns	41.0 ***	9.5 ***
	I × N		16.3***	22.0 ***	25.8 ***	4.5 **	8.7 ***	6.0 **	8.8 ***	9.1 ***
	Y × I × N		1.6 ns	2.1 ns	1.0 ns	0.5 ns	3.5 *	0.3 ns	0.5 ns	1.6 ns

CIF and MSF represent conventional irrigation and fertilization and micro-sprinkling fertigation, respectively. N0, N120, N180, and N240 represent the nitrogen application rate of 0, 120, 180, and 240 kg ha^−1^. ITN, inefficient tiller number; ITW, dry weight of the inefficient tillers; EN_-10_, number of ears with less than 10 grains; EN, ear number; GN, grain number; TGW, 1000-grain weight; GY, grain yield; and HI, harvest index. The different lowercase letters after values indicate significant difference at *p* < 0.05 among treatments. *, **, ***, and ns represent significant difference at *p* < 0.05, *p* < 0.01, *p* < 0.001, and *p* > 0.05, respectively.

**Table 2:** Effects of irrigation methods with different nitrogen application rates on the nitrogen absorption, accumulation, and distribution characteristics during the two growing seasons.

Year	Irrigation Methods	Nitrogen Application Rates	NA (kg ha^−1^)	NITM (kg ha^−1^)	NSM (kg ha^−1^)	NGM (kg ha^−1^)	NTM (kg ha^−1^)	NPA (kg ha^−1^)	CRPATG (%)	NHI (%)
2020–2021	CIF	N0	39.7 ± 2.4 e	0.1 ± 0.1 f	7.3 ± 1.1 f	42.1 ± 4.5 d	49.3 ± 5.4 d	9.6 ± 3.8 f	0.22 ± 0.07 c	0.85 ± 0.01 a
		N120	159.7 ± 7.8 cd	4.2 ± 0.9 d	46.2 ± 3.7 e	147.5 ± 11.1 c	193.7 ± 9.1 c	34.0 ± 9.5 e	0.23 ± 0.06 c	0.76 ± 0.03 c
		N180	188.4 ± 8.3 b	8.2 ± 1.6 b	78.4 ± 8.1 c	169.7 ± 0.9 b	248.0 ± 8.6 b	59.6 ± 4.0 cd	0.35 ± 0.02 b	0.68 ± 0.02 e
		N240	210.7 ± 11.9 a	11.1 ± 1.1 a	99.4 ± 3.7 a	173.8 ± 15.8 b	273.2 ± 10.3 a	62.5 ± 11.9 bc	0.36 ± 0.05 b	0.64 ± 0.01 f
	MSF	N0	35.4 ± 3.9 e	0.2 ± 0.3 f	7.3 ± 0.3 f	36.3 ± 5.6 d	43.6 ± 5.9 d	8.2 ± 2.3 f	0.22 ± 0.04 c	0.83 ± 0.02 a
		N120	150.0 ± 13.0 d	2.7 ± 0.5 e	40.7 ± 4.4 e	153.2 ± 5.0 c	194.0 ± 6.8 c	44.0 ± 9.5 de	0.29 ± 0.07 b	0.79 ± 0.02 b
		N180	170.0 ± 12.7 c	5.5 ± 0.9 cd	69.9 ± 6.1 d	179.5 ± 9.3 ab	249.4 ± 10.9 b	79.4 ± 8.2 ab	0.44 ± 0.04 a	0.72 ± 0.02 d
		N240	188.5 ± 11.8 b	6.4 ± 0.6 c	87.9 ± 5.0 b	191.6 ± 12.9 a	279.5 ± 16.4 a	91.0 ± 12.5 a	0.45 ± 0.04 a	0.69 ± 0.01 e
2021–2022	CIF	N0	63.4 ± 7.9 d	0.4 ± 0.1 f	15.3 ± 1.4 e	63.9 ± 5.5 d	79.2 ± 6.9 d	15.8 ± 1.0 d	0.25 ± 0.04 c	0.81 ± 0.00 a
		N120	165.8 ± 12.8 c	6.5 ± 0.3 d	53.9 ± 1.2 c	155.8 ± 4.5 c	209.6 ± 3.8 c	43.8 ± 9.0 c	0.28 ± 0.07 c	0.74 ± 0.01 c
		N180	234.0 ± 18.5 a	10.0 ± 0.5 b	88.9 ± 1.6 b	204.7 ± 6.3 b	293.6 ± 7.6 b	59.6 ± 15.5 b	0.29 ± 0.08 c	0.70 ± 0.00 e
		N240	246.5 ± 15.2 a	13.0 ± 2.2 a	103.2 ± 6.4 a	211.3 ± 10.9 b	314.5 ± 7.5 a	68.0 ± 11.2 b	0.32 ± 0.05 bc	0.67 ± 0.02 f
	MSF	N0	64.7 ± 9.0 d	0.5 ± 0.1 f	15.7 ± 2.4 e	67.0 ± 8.3 d	82.8 ± 10.5 d	18.1 ± 2.8 d	0.27 ± 0.04 c	0.81 ± 0.01 a
		N120	145.9 ± 14.4 c	3.8 ± 1.1 e	47.3 ± 3.8 c	158.0 ± 11.9 c	205.3 ± 15.6 c	59.4 ± 3.5 bc	0.38 ± 0.03 ab	0.77 ± 0.00 b
		N180	215.5 ± 11.1 b	7.5 ± 1.1 cd	84.2 ± 5.0 b	218.4 ± 18.0 ab	302.6 ± 19.1 ab	87.1 ± 12.1 a	0.40 ± 0.04 ab	0.73 ± 0.02 cd
		N240	218.3 ± 9.5 b	8.9 ± 1.6 bc	88.3 ± 4.9 b	223.5 ± 8.4 a	311.8 ± 12.6 ab	93.5 ± 13.7 a	0.42 ± 0.05 a	0.72 ± 0.01 de
ANOVA (F-value)	Y		72.1 ***	34.0 ***	32.7 ***	99.1 ***	120.2 ***	5.8 *	0.1 ns	0.3 ns
	I		19.8 ***	69.0 ***	21.5 ***	8.6 **	0.3 ns	29.6 ***	25.2 ***	33.2 ***
	N		554.6 ***	250.0 ***	783.8 ***	660.3 ***	1134.0 ***	105.8 ***	25.6 ***	235.0 ***
	Y × I		0.0 ns	0.0 ns	0.0 ns	0.1 ns	0.1 ns	0.3 ns	0.4 ns	0.1 ns
	Y × N		7.2 **	2.9 ***	2.6 ns	5.7 **	4.6 **	0.5 ns	4.2 *	12.6 ***
	I × N		2.5 ns	12.6 ***	4.3 *	1.8 ns	0.2 ns	4.3 *	2.3 ns	7.9 ***
	Y × I × N		0.2 ns	0.3 ns	0.3 ns	0.3 ns	0.5 ns	0.2 ns	0.0 ns	0.7 ns

CIF and MSF represent conventional irrigation and fertilization and micro-sprinkling fertigation, respectively. N0, N120, N180, and N240 represent the nitrogen application rate of 0, 120, 180, and 240 kg ha^−1^. NA, nitrogen accumulation at anthesis; NITM, nitrogen accumulation in ineffective tillers at maturity; NSM/NGM/NTM represent the nitrogen accumulation of straw, grain, and total plant at maturity, respectively; NPA is the nitrogen accumulation from post-anthesis to maturity; CRPATG is the post-anthesis nitrogen translation contribution to the grain N; and NHI, nitrogen harvest index. The different lowercase letters after values indicate significant difference at *p* < 0.05 among treatments. *, **, ***, and ns represent significant difference at *p* < 0.05, *p* < 0.01, *p* < 0.001, and *p* > 0.05, respectively.

**Table 3:** Effects of irrigation methods with different nitrogen application rates on the nitrogen use efficiency during the two growing seasons.

Year	Irrigation Methods	Nitrogen Application Rates	NPFP (kg/kg)	NAE (kg/kg)	NIUE (kg/kg)	NPE (kg/kg)
2020–2021	CIF	N0	—	—	100.0 ± 9.1 a	—
		N120	71.1 ± 2.4 a	30.1 ± 2.4 ab	44.1 ± 2.6 bc	25.8 ± 1.6 ab
		N180	50.0 ± 0.9 c	22.6 ± 2.9 c	36.3 ± 0.9 bc	21.2 ± 1.6 c
		N240	37.1 ± 0.3 e	16.6 ± 1.7 d	32.7 ± 2.3 c	17.4 ± 0.6 d
	MSF	N0	—	—	106.4 ± 16.1 a	—
		N120	72.3 ± 2.7 a	34.1 ± 2.5 a	44.8 ± 1.8 b	27.2 ± 1.4 a
		N180	53.9 ± 2.6 b	28.4 ± 1.4 b	38.9 ± 2.5 bc	24.8 ± 0.3 b
		N240	40.2 ± 0.4 d	20.6 ± 1.0 c	34.2 ± 1.6 c	21.0 ± 1.4 c
2021–2022	CIF	N0	—	—	44.0 ± 2.8 a	—
		N120	74.0 ± 2.9 b	45.2 ± 1.3 b	42.4 ± 1.4 ab	41.8 ± 4.2 b
		N180	54.9 ± 1.5 d	35.7 ± 0.7 c	33.7 ± 1.7 cd	29.9 ± 0.3 d
		N240	40.1 ± 0.8 f	25.6 ± 0.7 e	30.6 ± 1.3 d	26.2 ± 1.2 d
	MSF	N0	—	—	37.8 ± 3.9 bc	—
		N120	79.6 ± 2.2 a	53.5 ± 3.1 a	46.7 ± 2.4 a	53.2 ± 2.0 a
		N180	60.6 ± 1.5 c	43.2 ± 2.0 b	36.1 ± 1.4 c	35.4 ± 3.1 c
		N240	43.6 ± 1.0 e	30.5 ± 0.5 d	33.6 ± 1.3 cd	32.0 ± 1.1 c
ANOVA (F-value)	Y		63.3 ns	300.4 ns	122.7 ***	371.2 ***
	I		63.7 ***	59.3 ***	1.4 ns	50.4 ***
	N		3576.7 ***	227.7 ***	143.2 ***	111.8 ***
	Y × I		6.3 ns	4.0 ns	0.5 ns	8.3 **
	Y × N		9.2 ***	10.5 ***	104.0 ***	23.7 ***
	I × N		0.7 ns	1.2 ns	0.1 ns	0.1 ns
	Y × I × N		2.2 ns	1.4 ns	1.4 ns	1.8 ns

CIF and MSF represent conventional irrigation and fertilization and micro-sprinkling fertigation, respectively. N0, N120, N180, and N240 represent the nitrogen application rate of 0, 120, 180, and 240 kg ha^−1^. NPFP, nitrogen partial factor productivity; NAE, nitrogen agronomic efficiency; NIUE, nitrogen internal utilization efficiency; and NPE, nitrogen physiological efficiency. The different lowercase letters after values indicate significant difference at *p* < 0.05 among treatments. **, ***, and ns represent significant difference at *p* < 0.01, *p* < 0.001, and *p* > 0.05, respectively.

**Table 4:** Irrigation time, irrigation amount, and nitrogen application ratio of CIF and MSF in this study.

Treatments	Items	Before Sowing	Jointing Stage	Heading Stage	7 d After Anthesis	14 d After Anthesis	Total
CIF	Irrigation amount (mm)		75	75			150
	Nitrogen application ratio (%)	50	50				100
MSF	Irrigation amount (mm)		50	50	30	20	150
	Nitrogen application ratio (%)	40	30	15	10	5	100

CIF, conventional irrigation and fertilization; and MSF, micro-sprinkling fertigation.

## Data Availability

The raw data supporting the conclusions of this article will be made available by the authors on request.
